# The impact of a delayed consent model on trial recruitment - the cream study (children with eczema antibiotic management study)

**DOI:** 10.1186/1745-6215-16-S2-P199

**Published:** 2015-11-16

**Authors:** Victoria Shepherd, Emma Thomas-Jones, Katy Addison, Matthew Ridd, Christopher Butler, Kerenza Hood, Nick Francis

**Affiliations:** 1 Cardiff University, Cardiff, UK; 2 University of Bristol, Bristol, UK; 3 Oxford University, Oxford, UK

## Background

The CREAM Study is a randomised controlled trial that aims to address the uncertainty around the effect of oral and topical antibiotics on subjective and objective eczema severity in children with clinically infected eczema in primary care. Children were identified by GPs and referred to local CREAM research nurses. Research nurses arranged a baseline visit to the participant's home with 72 hours. Eligibility was re-confirmed at the visit and informed consent was obtained.

## Method

Screening logs recorded information about patients referred to the study team. Research nurses recorded details about whether patients referred to the study team were recruited and, if not, the reasons why.

## Results

The numbers of children recruited was lower than anticipated. 33% (58/171) of patients referred to the study by participating clinicians were not recruited as they either were identified by the research nurse as not meeting the study inclusion criteria 31% (18/58), did not wish to participate when contacted 21% (12/58), or due to difficulties in arranging a baseline visit within the study parameters 48% (28/58). The mean referral to recruitment interval was 1.27 days (range 0 - 4 days).

## Discussion

A number of recruitment issues were encountered in this study, including loss between identification and recruitment. The model of delayed consent, requiring re-confirmation of eligibility by research nurses within the days following identification in general practice, had a negative impact on participant recruitment. It resulted in loss between identification and recruitment, deterred some parents from participating and led to difficulties in expanding the study.

